# Pediatric phoniatry outpatient ward: clinical and epidemiological characteristics

**DOI:** 10.5935/1808-8694.20130029

**Published:** 2015-11-02

**Authors:** Mariana Lopes Fávero, Teresa Cristina Mendes Higino, Anna Paula Batista Pires, Patrick Rademaker Burke, Fernando Leite de Carvalho e Silva, Alfredo Tabith

**Affiliations:** a PhD in Sciences - ENT Department - FMUSP (Medical School of the University of São Paulo). Coordinator of the training programs in Phoniatry and Hearing Electrophysiology - DERDIC/PUCSP. Atending physician - HSPM-SP; b ENT specialist. Student of Hearing Electrophysiology - DERDIC/PUCSP; c MSc in Otorhinolaryngology - UNIFESP (Federal University of São Paulo). Student of Hearing physiology - DERDIC/PUCSP; d Specialist in Sleep Medicine - Department of Psycho-biology - UNIFESP. Fellow in Otoneurology - UNIFESP. Student of Hearing Electrophysiology - DERDIC/PUCSP; e ENT specialist. Coordinator of the Phoniatry and Otorhinolaryngology Department at DERDIC/PUCSP; f MSc in Communicaton Disorders - DERDIC/PUCSP. General Director of DERDIC/PUCSP. Atending physician at DERDIC/PUCSP and Professor at the Speech and Hearing School of the Catholic University of São Paulo - PUCSP. DERDIC/PUCSP

**Keywords:** child language, diagnosis, language development disorders

## Abstract

Children with language or learning impairment and normal hearing need phoniatric assessment to analyse various communication and development aspects targeting the differential diagnosis and therapeutic indications.

**Objective:**

Characterize clinical and epidemiological features of a pediatric population treated in a phoniatric outpatient clinic.

**Method:**

A cross-sectional historical cohort study (retrospective study) was performed involving 68 patients undergoing phoniatric consultation. Outcome measures were age, gender, source of referral for phoniatric consultation, phoniatric diagnosis, mean age at diagnosis, neonatal risks, family history of communication disorders and referrals.

**Results:**

70.58% were male and 29.42% female, mean age 6.85 ± 2.49 years. 63.23% from external services and 45.59% had no hearing diagnosis. 14 different diagnoses were performed: 50% had Cerebral Palsy, Specific Language Impairment and Pervasive Developmental Disorder. The difference between the average ages was statistically significant (F = 4.369 *p* = 0.00). 50% had a family history of communication disorders and 51.47% history of neonatal risk. 51.47% were referred for neurological consultation and 79.41% for therapies.

**Conclusion:**

The population seen was predominantly male, with more complex language development deviations probably due to multiple etiologies. Many of them had no hearing diagnosis.

## INTRODUCTION

Many children with language or learning disorders are referred to the ENT physician by schools, speech and hearing therapists, teachers, pediatricians and neurope-diatricians in order to rule out hearing loss^1^. That is so because hearing is one of the essential senses in human communication development, and any disorder anywhere in the auditory system^2^ may cause damages to this process^3^.

Notwithstanding, we are not always ready to deal with a complaint of learning and language development disorder when hearing is normal. In order to carry out the differential diagnosis of the problem, we need to consult a phoniatrician, who studies numerous aspects of a child's communication and development^4^. This careful clinical investigation during the medical consultation enables the physician to conceive diagnostic hypotheses and to indicate the best treatment for each case^5^.

Phoniatry is a field of otorhinolaryngology which manages human communication disorders, concentrating in voice, speech, language, hearing and swallowing^6^. The complexity of human communication justifies the intense array of possible diagnoses and requires a network of professionals, including physicians and non-physicians, for a proper diagnosis and to select the most adequate treatment. Thus, a Phoniatry Ward must have not only ENT physicians trained in phoniatric care, but also a number of other professionals who may take part in patient care, such as neurologists, psychiatrists, geneticists, speech and hearing therapists, psychologists and physical therapists, chosen considering the characteristics of the patient population.

This study was developed aiming at making a clinical and epidemiological characterization of the pediatric population seen at the Phoniatry Ward of our clinic. The authors hope to stimulate the creation of new phoniatric care centers, which are still rare in our country.

## METHOD

The study project was approved by the Ethics in Research Committee of our Institution (research protocol 57/10).

We did a cross-sectional historical cohort study, and we analyzed data from 68 patients examined by the team of phoniatricians of our institution. We included only those patients with a functional and/or etiological diagnosis established at the time of the study and we excluded the patients with ages equal to or older than 18 years.

The phoniatric consultation was structured in the form of a semi-open interview, in which the physician asks some questions and the patient, or companion, is free to report at will or to explain the understanding about the complaints. From this anamnesis we collected important data which helped in the diagnosis, such as: the child's relationship with the family and with the environment, neuropsychomotor development, feeding, school performance and personal and family's medical history.

Physical exam included playing with the child, using symbolic games, drawings, children books or jigsaw puzzles - since playing releases inhibitions and builds a space of trust between the physician and the child. We carried out a complete otorhinolaryngological exam and we studied the auditory and visual perceptive functions, general motor and oral functions, static and dynamic balances and spatial organization of the body and graphic planes - when the clinical manifestations required and the patient's age allowed.

The outcome measures used in the analysis were: patient age, gender, origin of the referral for the phoniatric consultation, phoniatric diagnosis, mean age at each diagnosis, neonatal risks, family relations concerning communication disorders and referrals made by phoniatricians.

The statistical analysis concerning the mean age at each diagnosis was carried out by means of the ANOVA test, used to compare the means from different populations, aiming at establishing sample variability^7^.

## RESULTS

Of the 68 patients, 48 (70.58%) were males and 20 (29.42%) were females, with mean age of 6.85 ± 2.49 years.

Insofar as the origin of referrals are concerned, 43 (63.23%) patients were referred for phoniatric consultation by external sources and 25 (36.76%) patients were already in our clinic's roster, either in otorhinolaryngological care or in therapies.

Of the 43 patients referred from other clinics, 31 (72.09%) were referred because of suspicion of hearing loss and were submitted to clinical, psychoacoustic, electrophysiological and electroacoustic tests pertaining to the diagnosis. Of the 31, only five (16.13%) really had hearing loss.

The 12 remaining patients who had been referred from outside the institution and the 25 patients who already were our patients, therefore, 54.41% of the total population of the study, already had had hearing assessments carried when they came to the phoniatric consultation and that was normal.

We defined 14 different phoniatric diagnoses in the population studied - which, together with the data of family past for communication disorders and neonatal risks are described on Table 1. The mean age of the patients in function of the phoniatric diagnosis may also be seen on Table 1. Comparing the mean ages of the patients for each diagnosis by the ANOVA test, we notice statistically significant differences among them (F = 4.369 *p* = 0.00).

**Table 1:** Diagnosis, age, family history concerning communication disorders and neonatal risks in the study group.

Diagnosis	Mean age	Number	Family history	Neonatal risks
1 - SLD	9.38 ± 2.68	11 (16.18%)	5	3 Mild fetal distress
2 - Language disorder without a defnitive etiology	4.3 ± 0.64	4 (5.89%)	3	1 Mild fetal distress
3 - ADHD	8.2 ± 1.70	4 (5.89%)	3	1 Mild fetal distress
				1 Case of gestational hypertension
4 - Pervasive development disorder	6.33 ± 0.72	9 (13.24%)	6	2 Light therapy
				1 Gestational hypertension
5 - Velopharyngeal dysfunction	6.3 ± 0.0	1 (1.47%)	-	Neonatal seizure
6 - Oral dyspraxia	7.15 ± 0.49	2 (2.94%)	-	2 Severe fetal distress
7 - Articulatory disorder	4.95 ± 0.21	2 (2.94%)	1	-
8 - Brain palsy	7.50 ± 2.69	14 (20.59%)	5	10 Prematurity
				4 Severe anoxia
9 - Hearing loss	4.56 ± 1.27	5 (7.35%)	2	1 Ototoxic drug during gestation; 1 Severe fetal distress
10 - Disfuence	7.85 ± 0.92	2 (2.94%)	2	1 Severe fetal distress
11 - Intelectual-cognitive defcit	8.33 ± 1.77	4 (5.89%)	1	2 Severe fetal distress
12 - Dysphonia	8.8 ± 0.0	1 (1.47%)	1	-
13 - Syndromes (4 Down syndrome, 2 velocardiofacial syndrome)	4.28 ± 0.91	6 (8.82%)	3	2 Prematurity 1 Cyanosis/Fallot's tetralogy
14 - Infectious origin (2 post-meningitis and 1 congenital toxoplasmosis)	4.4 ± 1.06	3 (4.41%)	2	1 Fetal distress 1 Neonatal anoxia

SLI: Specifc Language Impairment; ADHD: Attention Defcit Hyperactivity Disorder.

During the phoniatric assessment, 35 patients (51.47%) were referred to a neurological consultation and 31 (45.58%) were referred to auditory assessment, as per previously mentioned. Of these 31 patients, we had to do a psychoacoustic assessment in 12 (38.70%) for hearing diagnostic purposes, and in 19 (61.30%) we also did electrophysiological and electroacoustic assessment, under sedation, in order to establish the final hearing diagnosis.

**Graph 1:**
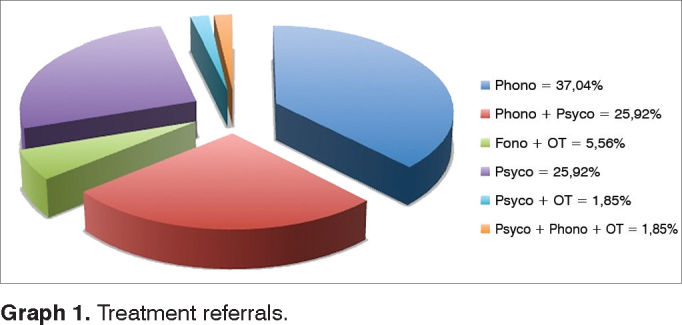
Treatment referrals.

After the phoniatric diagnosis, 54 (79.41%) patients were referred to treatments, according to Graph 1.

## DISCUSSION

Numerous diagnoses are possible in phoniatry, because of the disorders which may affect the organs and systems associated with human communication.

In our series, we found 14 types of disorders, mainly affecting males (70.58%), which is in agreement with literature data, indicating a greater prevalence of language disorders in this group^8^, ^9^. An array of hypothesis have been raised in order to try to explain the predominance of boys with communication disorders, including a greater vulnerability concerning situations which impair children development^10^, cerebral maturation changes, hormonal issues associated with testosterone levels, and social issues^9^.

For the phoniatric consultation, we had 43 (63.23%) patients coming from other services and 25 (36.76%) referred by professionals from our own institution. What stands out is the high number of patients - 31 of 43 - referred from outside, in other words, 45.60% of the total population in our study, referred for communication development disorders and still without a definitive auditory diagnosis, thus still having hearing loss as one of the hypothesis for the delay in language skills development. Of these 31, only five (16.13%) had hearing loss.

We believe such data is explained by the scarce number of specialized services of children auditory diagnosis serving the Brazilian Public Healthcare System (SUS), being psychoacoustic assessments carried out with specialized speech and hearing therapists and with time to observe the child's auditory behavior, or by means of electrophysiological and electroacoustic assessments, under sedation or general anesthesia, since these children are no longer so small as to sleep by themselves and, often times, as a reflex or as part of a communication delay manifestation, they do not collaborate with the necessary rest required for the objective tests.

We found a predominance of patients diagnosed with Cerebral Palsy, SLD (specific language disorder) and Invasive Development Disorder. These three entities represent 50% of the sample and are characterized by more severe situations of language impairment, which, probably reflects our situation as a reference center in phoniatric care; notwithstanding, it may also reflect our situation as a reference center for pediatric auditory diagnosis, since many of these patients have difficulties to undergo psychoacoustic assessments and objective tests in services which are not prepared for it.

Analyzing the mean age at diagnosis also stresses the need we have for specialized services in the care of these children. Patients with clinical manifestations of more complex or difficult language development disorders from the viewpoint of hearing loss differential diagnosis came to our clinic at significantly higher ages, as is the case of patients diagnosed with SLD, ADHD, Dyspraxia, Intellectual-Cognitive Deficit, and Cerebral Palsy. Patients with clearer clinical manifestations, such as patients with syndromes (Down or velocardiofacial) or infections (congenital toxoplasmosis or meningitis) came earlier for the diagnosis.

Positive family history for communication disorders is very common in patients with language disorders^8^ and this idea that language impairments are partially associated with genetic inheritance^11^ is not new, with numerous genes already described in cases of hearing loss^12^, SLD^13^, Pervasive Development Disorder^14^, ^15^ Intellectual-Cognitive Deficit^16^.

Neonatal stress is also among the risk factors for communication disorders^17^, either because of a greater incidence of hearing loss^18^, language and learning disor-ders^19^, ^20^, changes to psyche development^21^ or, still, changes to neurological development^17^.

In our sample, we had 50% of the population with a family history of communication disorder and 51.47% with a history of neonatal risk. On the other hand, communication disorders are also very frequent in populations without risks or family history, where it can reach up to 30% of the children in school age^22^ and may be associated with environmental factors, thus frequently making it multifactorial the genesis of communication disorders.

As far as referrals are concerned, 51.47% of the patients were referred during the phoniatry assessment process to neurological evaluation, aiming at clarifying the participation of neurological conditions in the clinical picture.

After having the diagnostic hypothesis, 79.41% of the patients were referred to speech and hearing therapy, psychological therapy, occupational therapy and/or physical therapy. Therefore, one phoniatry ward must be associated with a network of professionals able to provide treatment to patients with communication disorders, able to take over the treatment of referred patients and be willing to make constant contact with the phoniatrician and the other healthcare professional taking care of the patient. We believe that such multidisciplinary care - which must include clinical discussions among the healthcare professionals involved - is paramount for a proper patient development and proper support to the family and school.

## CONCLUSION

We may conclude that the population coming to us is mainly made up of males, clinically characterized by more complex disorders in their language development, and many of them are still missing a conclusive auditory diagnosis - probably of multifactorial etiology - and the patients' mean ages are statistically higher because of the complex diagnosis.

It is worth stressing the need for new phoniatric care centers in our country including, not only phoniatricians, but also a pediatric audiological diagnostic service and a multidisciplinary team of healthcare professionals.
